# Genetically Predicted Serum 25‐Hydroxyvitamin D Concentrations in Related to Type 2 Diabetes Mellitus: A Mendelian Randomization Study

**DOI:** 10.1002/edm2.70050

**Published:** 2025-07-09

**Authors:** Jin Yang

**Affiliations:** ^1^ Department of Laboratory Medicine Obstetrics and Gynecology Hospital of Fudan University, Shanghai Key Lab of Reproduction and Development, Shanghai Key Lab of Female Reproductive Endocrine Related Diseases Shanghai China

**Keywords:** 25‐hydroxyvitamin D, multivariable Mendelian randomization, type 2 diabetes mellitus, univariable Mendelian randomization

## Abstract

**Background:**

In several observational studies, vitamins B6, B9, B12, C and 25‐hydroxyvitamin D[25(OH)D] concentrations were associated with type 2 diabetes mellitus (T2DM). Although vitamins play a role in the development of type 2 diabetes mellitus (T2DM), their associations remain unclear.

**Objective:**

This study employed Mendelian randomisation (MR) to explore the causal relationships between circulating concentrations of vitamins B6, B9, B12, C, 25‐hydroxyvitamin D and T2DM.

**Methods:**

Single‐nucleotide polymorphisms (SNPs) linked to vitamin B6, vitamin B9, vitamin B12, vitamin C and 25(OH)D levels were used as instrumental variables (IVs) in this study. We have two outcomes related to T2DM derived from two genome‐wide association studies (GWAS). The first study, referenced by PMID: 3417140, encompasses a cohort of 406,831 individuals of European descent. The second study, identified by PMID: 29892013, includes a sample size of 468,298 Europeans.

**Results:**

Both univariable Mendelian randomization (UVMR) and multivariable Mendelian randomization (MVMR) analyses demonstrate that genetically predicted elevated levels of serum 25(OH)D are consistently associated with a reduced risk of T2DM. In the UVMR analyses, A 1‐SD increase in genetically predicted serum 25(OH)D levels, the inverse‐variance weighted (IVW) *p* = 3.8 × 10^−7^, *p*_*fdr*_ = 7.6 × 10^−7^, the odds ratio(OR) of T2DM (GCST90013942) was 0.67, 95% confidence interval (CI): 0.57–0.78. Furthermore, a 1‐SD increase in genetically predicted serum 25(OH)D levels was associated with an OR of 0.987 for T2DM (GCST90029024), the IVW *p* = 1.1 × 10^−4^, *p*_*fdr*_ = 1.1 × 10^−4^ with a 95% CI of 0.981–0.994. In the MVMR analyses, genetically predicted higher serum 25(OH)D levels were associated with a decreased risk of T2DM by the IVW *p* = 1.2 × 10^−5^, *p*_*fdr*_ = 5.9 × 10^−5^ in GCST90013942 and IVW *p* = 4.9 × 10^−4^, *p*_*fdr*_ = 2.5 × 10^−3^ in GCST90029024. In contrast, levels of vitamins B6, B9, B12, and C did not domenstrate a significant association with T2DM.

**Conclusion:**

Our research reveals that higher circulating serum 25(OH)D levels reduce the possibility of T2DM.

## Graphical abstract

Recent studies have demonstrated an association between the concentrations of vitamins B6, B9, B12, C, and 25‐hydroxyvitamin D and the incidence of type 2 diabetes mellitus. This study utilized Mendelian randomization (MR) to investigate the causal relationships between circulating levels of these vitamins and T2DM. Single nucleotide polymorphisms associated with levels of vitamins B6, B9, B12, C, and 25‐hydroxyvitamin D were employed as instrumental variables in this study. Both univariable Mendelian randomization (UVMR) and multivariable Mendelian randomization (MVMR) analyses indicate that genetically predicted higher serum levels of 25‐hydroxyvitamin D are consistently linked to a decreased risk of type 2 diabetes mellitus. Our study indicates that elevated levels of circulating serum 25‐hydroxyvitamin D are associated with a decreased risk of developing type 2 diabetes mellitus.

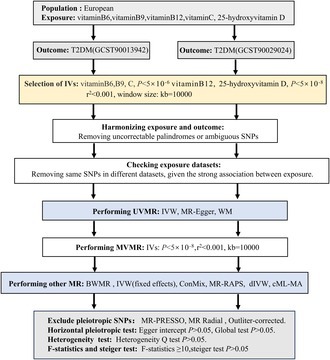

## 1 Introduction

Type 2 diabetes mellitus (T2DM) is a chronic metabolic disorder marked by sustained hyperglycemia, which, over time, leads to considerable damage to peripheral nerves, ocular structures, renal function, and cardiovascular system [1, 2]. This condition predominantly occurs in adults and results from the body's insulin resistance or inadequate insulin production [3, 4]. Type 2 diabetes mellitus(T2DM) constitutes approximately 90% of all diabetes cases and is highly prevalent. According to recent data from the International Diabetes Federation (IDF), the global prevalence of diabetes among individuals aged 20–79 years was estimated at 10.5% in 2021, equating to approximately 536.6 million people. This prevalence is projected to rise to 12.2%, or 783.2 million individuals, by 2045 [5]. Type 2 diabetes mellitus (T2DM) arises from a multifaceted interaction among genetic, epigenetic and environmental determinants [6, 7, 8]. Understanding disparities in risk factor profiles and the burden of diabetes across different populations is crucial for informing strategies aimed at effectively managing diabetes risk factors, particularly within the context of multiple and complex determinants [9].

Several studies have demonstrated correlations between vitamins and their potential benefits in the management of diabetes and its complications. Micronutrients are critical to the development and complications of diabetes. Recent studies indicate that deficiencies in vitamins B9 and B12 within certain populations may potentially increase the risk of diabetic retinopathy [10, 11]. Research findings suggest that vitamin C supplementation may have a positive impact on certain complications of diabetes, particularly diabetic foot ulcers [12, 13]. Vitamin D has emerged as a potential determinant of the risk of T2DM, and vitamin D supplementation has been proposed as a possible intervention to reduce this risk [14, 15]. A meta‐analysis demonstrated a significant inverse association between circulating 25‐hydroxyvitamin D [25(OH)D] levels and the risk of T2DM in diverse populations [16]. The majority of vitamin D circulating in the body is synthesised in the skin from 7‐dehydrocholesterol in the presence of sunlight [17]. Initially, vitamin D is converted into 25‐hydroxyvitamin D[25(OH)D] in the liver, and then into 1,25‐dihydroxyvitamin D in the kidneys [18]. 25‐hydroxyvitamin D[25(OH)D] is the primary circulating form of vitamin D and is an excellent biomarker for assessing overall vitamin D levels [19]. According to the US Endocrine Society, vitamin D deficiency is characterised by a serum 25(OH)D level of ≤ 50 nmol/L(20 ng/mL), while insufficiency is defined as a level of 52.5–72.5 nmol/L(21–29 ng/mL), and sufficiency commences at a level of 75 nmol/L (30 ng/mL), with the most beneficial levels falling between 90 and100 nmol/L (36–40 ng/mL) [20]. Despite a systematic review examining the effect of vitamin D status on insulin resistance and glycemic control in individuals with prediabetes [21], A subsequent study found no benefit of vitamin D in improving insulin resistance. The existing research on the relationship between vitamins and T2DM yields conflicting results. T2DM and genetically predicted vitamin levels were examined using Mendelian randomization (MR) analysis.

MR analysis aims at investigating causal relationships between modifiable risk factors and diseases. The use of genetic variations, particularly SNPs, as instrumental variables (IVs) in MR allows for the estimation of exposure effects while mitigating concerns related to reverse causality and confounding factors. The concept of pleiotropy, wherein genetic variations have multiple effects, is also a consideration in MR studies. We conducted MR methods to analyse associations between vitamins and T2DM. Specifically, our objective was to investigate the potential causal relationship between vitamin D (25‐hydroxyvitamin D) levels and T2DM. The classic inverse variance weighted (IVW) method demonstrates a stable detection performance, whereas the recently developed MR methods each offer unique benefits. Therefore, we employed the new MR methods to examine its detection efficacy.

## 2 Study Design

**FIGURE 1:**
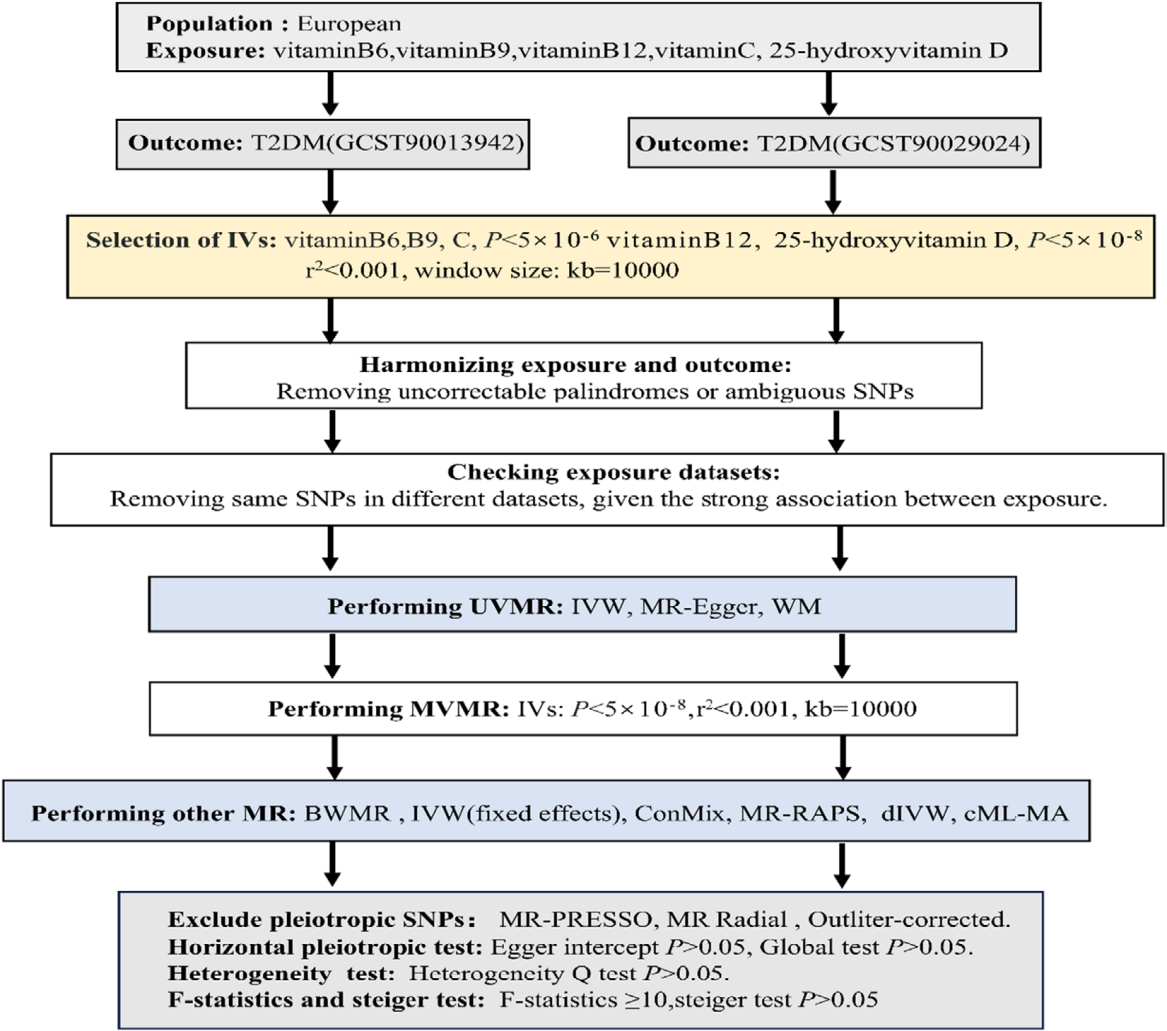
Flow chart for instrumental variables selection and MR analyses.

The three principal hypotheses of MR are articulated as follows: (1) The correlation hypothesis postulates a significant association between SNPs and exposure variables. (2) The independence assumption asserts that SNPs are independent of any potential confounding variables. (3) The exclusivity hypothesis proposes that SNPs influence outcomes solely through exposure variables. We identified robust instrumental variables (IVs) for both univariable Mendelian randomization (UVMR) and multivariable Mendelian randomization (MVMR) analyses. To satisfy the independence assumption, it is imperative to eliminate confounding variables, which requires the exclusion of IVs that are associated with these confounders. The relationship between IVs and phenotypes can be examined utilising the LDLink platform (https://ldlink.nih.gov) [22] Tables S1 and S2. We employed pleiotropic and heterogeneity tests to assess the validity of the IVs. We respectively employed UVMR and MVMR analyses to investigate the associations between serum levels of vitamins B6, B9, B12, C, 25(OH)D and T2DM (two outcomes). Then, several developed MR methods were used to study the relationship between 25(OH)D and T2DM (two outcomes). Summary data from published GWAS were employed, with all included studies having received approval from relevant ethics review committees and having obtained informed consent from participants. The research design is illustrated in Figure 1.

### 2.1 Data Sources

In our study, we utilised GWAS datasets on vitamin B6, vitamin B9, and vitamin C obtained from the UK Biobank. The GWAS data for vitamin B6 encompassed a European cohort consisting of 64,979 individuals. For vitamin B9, the GWAS data was derived from a European population, comprising 10,049 cases and 450,302 controls. The vitamin C GWAS data was derived from a European population, including 28,536 cases and 307,055 controls. The 25(OH)D level GWAS data was sourced from the study by Manousaki et al. (2020). The serum 25(OH)D levels in the cohort ranged from 10 to 130 nmol/L, with a median of 69.9 nmol/L, a mean of 70 nmol/L, and a standard deviation (SD) of 34.7 nmol/L. The vitamin B12 GWAS data was obtained from the FinnGen study, which included 3694 cases and 393,684 controls from a European population. Additionally, GWAS datasets for T2DM were sourced from studies conducted by Mbatchou et al. (2021) (PMID: 34017140) and Loh et al. (2018) (PMID: 29892013). Outcome data (Type 2 diabetes mellitus GWAS data) were sourced from Mbatchou J (2021) (PMID: 34017140) and Loh PR (2018) (PMID: 29892013). Mbatchou J's study involved a European cohort of 406,831 individuals, while Loh PR's research encompassed a slightly larger European population of 468,298 participants. Details of the GWAS datasets are displayed in Table 1. The overlap rate of our datasets and Type I error rate were calculated using the website available at https://sb452.shinyapps.io/overlap/. The analyses revealed that the overlap rate across all datasets was below 10%, and the Type I error rate was less than 0.05, suggesting that our findings were both robust and reliable [23, 24].

**TABLE 1:** GWAS data source of exposure and outcomes.

Exposure	Trait	Consortium	Population	Sex	Sample size (case/control)	nSNPs	Author	Year
ieu‐b‐4808	25 Hydroxyvitamin D level	IEU	European	Males and females	441,291	16,668,957	Manousaki	2020
ukb‐b‐3563	Vitamin B9	UKBiobank	European	Males and females	10,049/450,302	9,851,867	Ben Elsworth	2018
ukb‐b‐7864	Vitamin B6	UKBiobank	European	Males and females	64,979	9,851,867	Ben Elsworth	2018
ukb‐a‐461	Vitamin C	UKBiobank	European	Males and females	28,536/307,055	10,894,596	Neale	2017
finngen_R10_B12_DEF	Vitamin B12	FinnGen	European	Males and females	3694/393,684	19,345,434	—	2023
**Outcome**								
GCST90013942	Type 2 diabetes	EBI‐GWAS‐catalogue	European	Males and females	406,831	11,038,957	Mbatchou J	2021
GCST90029024	Type 2 diabetes	EBI‐GWAS‐catalogue	European	Males and females	468,298	11,973,400	Loh PR	2018

### 2.2 The Selection of Instrumental Variables

IVs must be correlated with exposure factors to fulfil the relevance assumption, the initial of three fundamental assumptions. In the UVMR analyses, IVs were selected for vitamin B6, vitamin B9 and vitamin C based on *p*_*GWAS*_ < 5 × 10^−6^, for vitamin B12 and 25(OH)D based on *p*_*Gwas*_ < 5 × 10^−8^. In the MVMR analyses, the IVs met the threshold of *p*_*GWAS*_ < 5 × 10^−8^. By linkage disequilibrium (LD) clumping, independent SNPs for the exposures were found using *r* ^2^ < 0.001 and an allele distance exceeding 10,000 kilobases. From the GWAS datasets of outcomes, SNPs with minor allele frequencies of less than 0.01 were excluded from the analysis of SNPs and their associated statistics. Using proxy SNPs exhibiting a high correlation coefficient (*r* ^2^ > 0.8), we addressed missing SNPs. Additionally, we harmonised the data by excluding all palindromic SNPs. For the second MR assumption, we queried each IV and its proxy traits using the LDLink package (http://cran.r‐project.org/web/packages/LDlinkR/index.html), and eliminated SNPs associated with confounding traits at *r* ^2^ > 0.8. We employed the MR‐Radial method to assess whether the SNPs satisfy the established criteria, subsequently eliminating the outlier SNPs identified by the Egger and IVW methods.

### 2.3 The Strength of Instrumental Variables

Following the calculation of the proportion of phenotypic variation explained by all SNPs, we calculated the *F*‐statistic to assess the effectiveness of our IVs in elucidating phenotypic variation, using the formula $F\;\text{power}={\left(\beta /\mathrm{Se}\right)}^{2}$ [25, 26]. An *F*‐statistic greater than 10 indicates a significant ability to explain phenotypic variation [27]. We utilised the tool available at https://sb452.shinyapps.io/power/ to calculate the power [28] for each exposure. Power is a statistic that represents the probability of detecting true effects in a study. A higher value of power indicates a reduced likelihood of committing Type II errors [29]. *R* ^2^ represents the proportion of phenotypic variation that is explained (Tables 2 and 4). In this study, the IVs chosen are mutually independent, and the explanatory impact of each IV on exposure variation is likewise independent. *R* ^2^ is calculated as the cumulative sum of the exposure proportions explained by each IV. $R^{2}=2\times \mathrm{MAF}\times \left(1-\mathrm{MAF}\right)\times \beta^{2}$ [30]. The power for the vitamin C GWAS dataset (ukb‐a‐461) in relation to T2DM(GCST90013942) was 48%, whereas the power for the other datasets exceeding 48%.

**TABLE 2:** Pleiotropy and heterogeneity test results for five vitamins and T2DM (GCST90013942).

Exposure	IVs	*R* ^*2*^ (%)	*F*‐statistic	Egger intercept	*p* ^*a*^	Cochran's *Q*	Q‐df	*p* ^*b*^
Vitamin C	12	6.5 × 10^−3^	22.6	−7.2 × 10^−4^	0.60	8.7	11	0.65
Vitamin B9	10	1.5 × 10^−3^	30.4	−0.02	0.30	11	9	0.27
Vitamin B6	14	0.48	22.7	4.0 × 10^−3^	0.67	8.7	13	0.8
Vitamin B12	4	4.81	42.9	0.02	0.57	1.4	3	0.7
25‐Hydroxyvitamin D	141	0.91	33.4	−5.0 × 10^−3^	0.11	131.1	140	0.69

*Note:* *p* ^*a*^ is the value of *p* for MR—Egger intercept. *p* ^*b*^ is the value of *p* for heterogeneity tests by performing inverse‐variance weighted method. *R* ^2^ is proportion of variance in exposure variable explained by SNPs.

**TABLE 3:** MR‐PRESSO results for five vitamins and T2DM (GCST90013942).

Exposure	MR‐PRESSO	MR‐radial (Egger)	MR‐radial (IVW)	Confounding factors	Outliter‐corrected SNPs	*β*	Se	*p* ^*a*^	*p* ^*b*^
Vitamin C	2	3	3	0	12	1.41	0.84	0.66	0.19
Vitamin B9	0	0	1	0	10	2.88	2.22	0.31	0.02*
Vitamin B6	0	0	2	0	14	3.28	0.09	0.81	0.01*
Vitamin B12	3	1	0	1	4	−0.12	0.02	0.78	0.91
25‐Hydroxyvitamin D	0	17	25	2	141	−5.25	0.08	0.71	5.6 × 10^−7^ *

*Note:* *p* ^a^ is the value of p for the global test performed by the MR–PRESSO method to detect potential horizontal pleiotropy. *p* ^*b*^ is the value of *p* for MR–PRESSO analysis after outlier correction.

* *p* < 0.05, the result is statistically significant.

**FIGURE 2:**
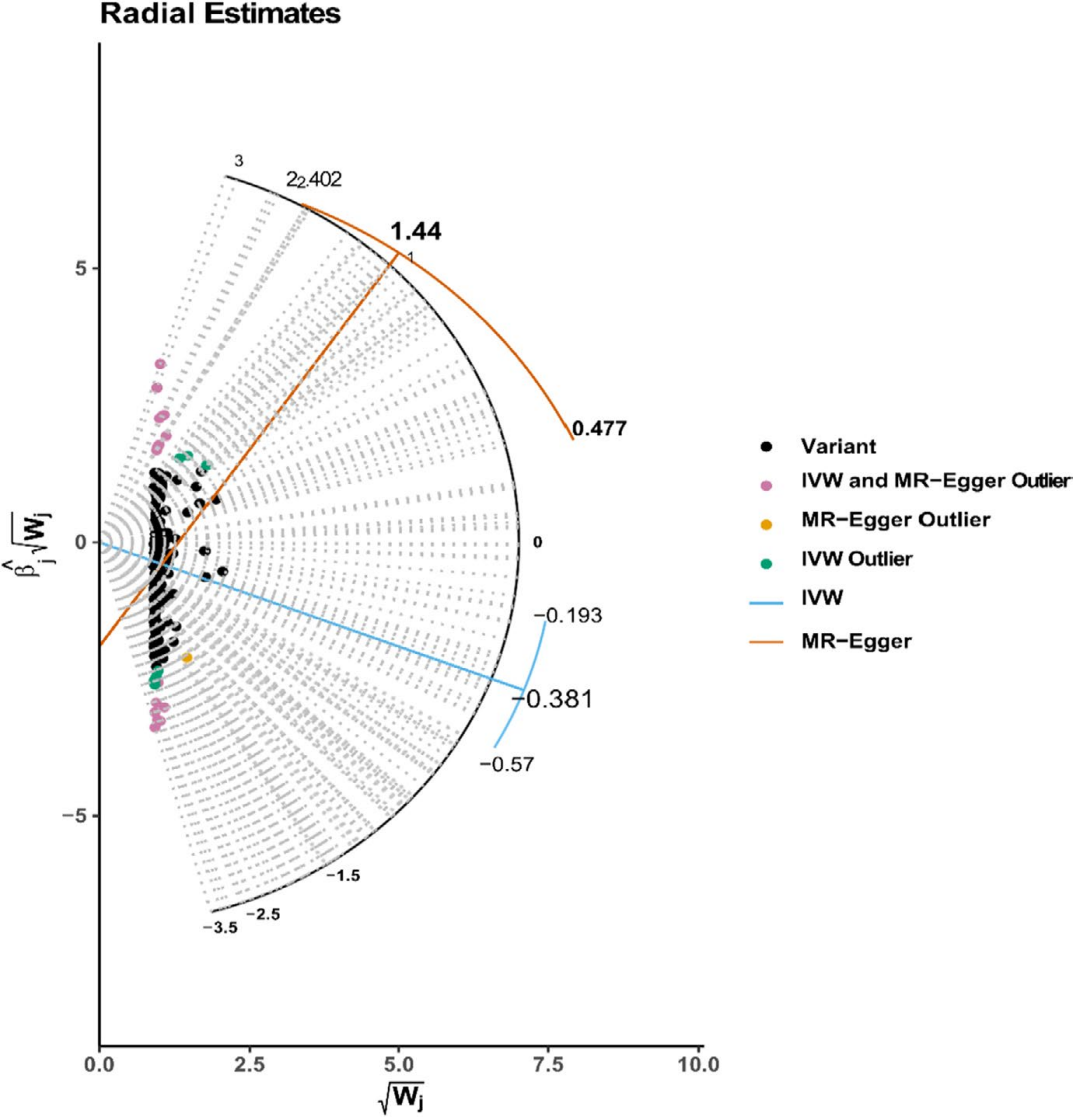
MR‐Radial plot of 25‐hydroxyvitamin D and T2DM (GCST90013942).

In the UVMR analyses, IVs were selected for vitamin B6, vitamin B9, and vitamin C based on *p*_*GWAS*_ < 5 × 10^−6^, and for vitamin B12 and 25(OH)D based on *p*_*GWAS*_ < 5 × 10^−8^. In the study of T2DM (GCST90013942), we identified and excluded two outliers (rs2476601, rs1990760) related to the confounding traits in vitamin B12 exposure. Additionally, one outlier (rs74203920) was identified using the MR‐Radial Egger method [31], and two outliers (rs1801222, rs28407950) were identified using the MR‐PRESSO method, resulting in a total of four IVs retained. For vitamin C exposure, two outliers (rs6679804, rs73105763) were removed using the MR‐Radial IVW (inverse‐variance weighted) method, and two outliers (rs11879199, rs2442736) were excluded using the MR‐PRESSO method, leaving a total of 12 IVs. In the analysis of vitamin B9 exposure, one outlier (rs12325280) identified by the MR‐radial IVW method was excluded, resulting in 10 IVs remaining. For vitamin B6 exposure, two outliers (rs155599, rs3745438) were removed using the MR‐radial IVW method, leaving 14 IVs. For 25(OH)D exposure, two outliers (rs1047891, rs11591147) associated with the confounding traits were excluded, which included 17 outliers identified by the MR‐radial Egger method and 25 outliters identifed by the MR‐radial IVW method, leaving a total of 141 IVs (Tables 2 and 3 and Table S3). MR‐Radial plot is presented in Figure 2.

In the study of T2DM (GCST90029024), we identified and excluded three outliers (rs12927355, rs151234, rs74203920) associated with confounding traits. Additionally, two outliers (rs2476601, rs28407950) were excluded using the MR‐PRESSO method, resulting in five IVs remaining for vitamin B12 exposure. For vitamin C exposure, two outliers (rs1022201, rs1187919) were removed using the MR‐radial IVW method, and one outlier (rs67659273) was excluded using the MR‐radial Egger method, leaving a total of 17 IVs. In the analysis of vitamin B9 exposure, one outlier (rs10771098) was removed using the MR‐radial IVW method, resulting in 11 IVs remaining. During the analysis of vitamin B6 exposure, one outlier (rs3745438) identified by the MR‐Radial IVW method was excluded, resulting in the retention of 14 IVs. For the case of 25(OH)D exposure, we excluded two outliers (rs1047891, rs11591147) associated with confounding traits. Two outliers (rs3817588, rs3761077) were identified by the MR‐PRESSO method, along with 27 outliers identified by the MR‐Radial Egger method, and 34 outliers identified by the MR‐Radial IVW method, leaving a total of 121 IVs (Tables 4 and 5 and Table S4).

**TABLE 4:** Pleiotropy and heterogeneity test results for five vitamins and T2DM (GCST90029024).

Exposure	IVs	*R* ^*2*^ (%)	*F*‐statistic	Egger intercept	*p* ^*a*^	Cochran's *Q*	Q‐df	*p* ^*b*^
Vitamin C	17	9.5 × 10^−3^	23.3	−1.1 × 10^−5^	0.98	18.9	16	0.28
Vitamin B9	11	1.7 × 10^−3^	31.0	−3.1 × 10^−4^	0.59	10.7	10	0.39
Vitamin B6	16	0.56	23.3	2.6 × 10^−5^	0.94	10.7	15	0.77
Vitamin B12	5	7.20	49.1	9.5 × 10^−4^	0.08	3.6	4	0.46
25‐Hydroxyvitamin D	121	0.79	32.5	−1.9 × 10^−4^	0.13	123.7	120	0.39

*Note:* *p* ^*a*^ is the value of *p* for MR—Egger intercept. *p* ^*b*^ is the value of *p* for heterogeneity tests by performing inverse‐variance weighted method. *R* ^2^ is proportion of variance in exposure variable explained by SNPs.

**TABLE 5:** MR‐PRESSO results for five vitamins and T2DM (GCST90029024).

Exposure	MR‐PRESSO	MR‐Radial(Egger)	MR‐Radial(IVW)	Confounding factors	Outliter‐corrected SNPs	*β*	Se	*P* ^*a*^	*P* ^*b*^
Vitamin C	0	1	2	0	17	0.95	0.03	0.23	0.36
Vitamin B9	0	0	1	0	11	2.39	0.08	0.42	0.04*
Vitamin B6	0	0	1	0	16	2.85	< 0.01	0.77	0.01*
Vitamin B12	2	0	0	3	5	1.42	< 0.01	0.44	0.23
25‐Hydroxyvitamin D	2	28	34	2	121	−3.86	< 0.01	0.39	1.8 × 10^−4^ *

*Note:* *p* ^a^ is the value of *p* for the global test performed by the MR–PRESSO method to detect potential horizontal pleiotropy. *p* ^b^ is the value of p for MR–PRESSO analysis after outlier correction.

* *p* < 0.05, the result is statistically significant.

Multiple mechanisms are implicated in the regulation of T2DM pathogenesis through the action of vitamin D. Among the SNPs selected as IVs in our study on vitamin D exposure, some have been previously reported in GWAS. The genetic variant rs3814995 has been documented to be associated with serum 25(OH)D levels [32]. Additionally, SNPs rs2207132, rs3814995, rs4364259, rs9861009, and rs11060406 have been reported to correlate with serum 25(OH)D levels in another GWAS [33]. An analysis of 35 blood and urine biomarkers within a cohort from the UK Biobank revealed a significant association between the genetic variant rs12128071 and vitamin D levels [34]. Furthermore, the genetic variant rs2519093 has been identified to correlate with insulin receptor levels, as demonstrated by a whole genome sequence study of the plasma proteome in Black adults [35]. Moreover, SNPs rs2306390, rs4364259, rs7314285, and rs9861009 have been identified as being associated with 25(OH)D levels in a study involving cross‐ancestry analyses that identified novel genetic loci linked to 25(OH)D [36].

### 2.4 Statistical Analysis

As part of the UVMR analyses, we utilised three MR methods: inverse‐variance weighted (IVW) [37], MR‐Egger [38], and weighted median (WM) [39]. The inverse‐variance weighted (IVW) method is based on the assumption that all SNPs function as valid IVs without exhibiting directional pleiotropy. This method synthesises the Wald ratios of genetically causal effects associated with each SNP, making the IVW method the primary analytical technique for estimating power and precision, as it offers the maximum power and precision.

We employed the MR‐PRESSO (Mendelian Randomization Pleiotropy RESidual Sum and Outlier) method to produce causal association estimates that are adjusted for outliers [40]. This procedure necessitates the exclusion of one or more pleiotropic marginal single SNP, followed by a re‐execution of the MR analysis. To ascertain whether the observed causal estimates were influenced by a reverse causal relationship, we utilised the Steiger test [41]. Additionally, we conducted heterogeneity, pleiotropy, and sensitivity analyses to evaluate potential biases arising from heterogeneity and pleiotropy. The Cochran's *Q* test [42] was employed to assess the heterogeneity among IVs. The leave‐one‐out approach was employed to assess the impact of individual SNPs on the overall MR estimation. If any SNP was identified as having a significant effect on the overall MR estimate, it was excluded from the analysis, and the datasets were subsequently re‐evaluated.

Given the correlational characteristics of the five vitamin biomarkers identified in the UVMR analyses, we conducted MVMR analyses [43, 44] to elucidate the primary factors influencing the causal relationship between vitamins and T2DM. IVs must be significantly associated with at least one exposure for one MVMR analysis to be valid. In each MVMR analysis, we identified the intersection of the five vitamin IVs to thoroughly assess the collective impact of these variables on the outcome.

## 3 Results

In the MVMR analyses, we identified 114 SNPs as IVs in the study of T2DM (GCST90013942) (Table 6 and Table S5), with IVs meeting *p*_*GWAS*_ < 5 × 10^−8^. The F‐statistic was computed for each of the 114 SNP groups, revealing that the SNPs associated with 25(OH)D exhibited values exceeding 10. This finding suggested the absence of bias due to weak IVs in this study. For five exposures of vitamins, we employed the False Discovery Rate (FDR) correction [45] to manage the false positive error rate in the context of multiple comparisons. The MVMR analysis indicated that genetically predicted serum 25(OH)D levels exhibit a significant causal effect on T2DM. The IVW (*p* = 1.2 × 10^−5^, *p*_*fdr*_ = 5.9 × 10^−5^), Egger (*p* = 8.1 × 10^−6^, *p*_*fdr*_ = 4.0 × 10^−5^), Lasso [46] (*p* = 5.9 × 10^−9^, *P*_*Fdr*_ = 2.9 × 10^−8^), and the Median (*p* = 1.6 × 10^−4^, *p*_*fdr*_ = 7.9 × 10^−4^). The effect size (*β* = −0.58) suggested a negative correlation between serum 25(OH)D levels and T2DM risk. It indicated higher serum 25(OH)D levels were associated with a decreased risk of T2DM. In contrast, genetically predicted vitamins B12, C and B9 did not demonstrate a significant causal relationship with T2DM risk. Notably, vitamin B6 demenstrated a significant effect on T2DM risk only when assessed using the Median method (*p* = 0.01, *p*_*fdr*_ = 0.02), while the IVW, Egger, and Lasso methods did not reveal any significant causal effects.

**TABLE 6:** MVMR of five vitamins on T2DM (GCST90013942).

Exposure	nSNPs	Method	*β*	Se	*p*	*p*_*fdr*_
Vitamin B12	114	IVW	0.04	0.04	0.39	0.49
	114	Egger	< −0.01	0.06	0.99	0.99
	114	Lasso	0.03	0.03	0.43	0.48
	114	Median	0.05	0.04	0.26	0.44
25‐hydroxyvitamin D	114	IVW	−0.58	0.13	1.2 × 10^−5^ *	5.9 × 10^−5^ *
	114	Egger	−0.59	0.13	8.1 × 10^−6^ *	4.0 × 10^−5^ *
	114	Lasso	−0.61	0.10	5.9 × 10^−9^ *	2.9 × 10^−8^ *
	114	Median	−0.59	0.16	1.6 × 10^−4^ *	7.9 × 10^−4^ *
Vitamin C	114	IVW	0.83	1.91	0.67	0.67
	114	Egger	0.94	1.91	0.62	0.78
	114	Lasso	1.05	1.48	0.48	0.48
	114	Median	0.49	1.95	0.79	0.79
Vitamin B9	114	IVW	5.19	4.24	0.22	0.37
	114	Egger	5.14	4.24	0.23	0.38
	114	Lasso	2.99	3.36	0.37	0.48
	114	Median	−1.58	4.48	0.72	0.80
Vitamin B6	114	IVW	0.48	0.25	0.05	0.13
	114	Egger	0.47	0.25	0.06	0.15
	114	Lasso	0.47	0.20	0.02*	0.05
	114	Median	0.68	0.25	0.01*	0.02*

* *p* < 0.05, the result is statistically significant.

Meanwhile, we identified 133 SNPs as IVs in the study of T2DM (GCST90029024) (Table 7 and Table S6), with IVs meeting *p*_*GWAS*_ < 5 × 10^−8^. The MVMR analysis also indicated that genetically predicted serum 25(OH)D concentrations have a significant causal effect on T2DM. The IVW (*p* = 4.9 × 10^−4^, *p*_*fdr*_ = 2.5 × 10^−3^), Egger (*p* = 3.7 × 10^−4^, *p*_*fdr*_ = 1.9 × 10^−3^), Lasso (*p* = 3.3 × 10^−6^, *p*_*fdr*_ = 1.6 × 10^−5^), and Median (*p* = 1.2 × 10^−4^, *p*_*fdr*_ = 6.1 × 10^−4^) results indicated that the effect size (*β* = −0.02) suggested a negative correlation between serum 25(OH)D concentrations and T2DM risk. It indicated higher serum 25(OH)D levels were associated with a decreased risk of T2DM. In contrast, genetically predicted vitamins B12 and C did not demonstrate a significant causal relationship with T2DM risk. Notably, vitamin B9 (*p* = 2.4 × 10^−3^, *p*_*fdr*_ = 3.3 × 10^−3^) and vitamin B6 (*p* = 2.0 × 10^−4^, *p*_*fdr*_ = 5.1 × 10^−4^) showed a significant effect on T2DM risk only when assessed using the Lasso method, while the IVW, Egger, and Median methods did not reveal any significant causal effects.

**TABLE 7:** MVMR of five vitamins on T2DM (GCST90029024).

Exposure	nSNPs	Method	*β*	Se	*p*	*p*_*fdr*_
Vitamin B12	133	IVW	−1.9 × 10^−4^	< 0.01	0.92	0.94
	133	Egger	< −0.01	< 0.01	0.43	0.54
	133	Lasso	4.6 × 10^−4^	< 0.01	0.74	0.74
	133	Median	−4.1 × 10^−4^	< 0.01	0.81	0.81
25‐hydroxyvitamin D	133	IVW	−0.02	0.01	4.9 × 10^−4^ *	2.5 × 10^−3^ *
	133	Egger	−0.02	0.01	3.7 × 10^−4^ *	1.9 × 10^−3^ *
	133	Lasso	−0.02	< 0.01	3.3 × 10^−6^ *	1.6 × 10^−5^ *
	133	Median	−0.02	0.01	1.2 × 10^−4^ *	6.1 × 10^−4^ *
Vitamin C	133	IVW	0.01	0.08	0.94	0.94
	133	Egger	0.01	0.08	0.91	0.91
	133	Lasso	0.07	0.06	0.26	0.33
	133	Median	0.08	0.08	0.32	0.39
Vitamin B9	133	IVW	0.41	0.19	0.03*	0.07
	133	Egger	0.41	0.19	0.03*	0.07
	133	Lasso	0.39	0.13	2.4 × 10^−3^ *	3.3 × 10^−3^ *
	133	Median	0.39	0.18	0.03*	0.06
vitamin B6	133	IVW	0.02	0.01	0.11	0.19
	133	Egger	0.02	0.01	0.12	0.20
	133	Lasso	0.03	0.01	2.0 × 10^−4^ *	5.1 × 10^−4^ *
	133	Median	0.02	0.01	0.05	0.08

* *p* < 0.05, the result is statistically significant.

In the UVMR analyses, IVs were selected for vitamin B6, vitamin B9, and vitamin C based on *p*_*GWAS*_ < 5 × 10^−6^, and for vitamin B12 and 25(OH)D based on *p*_*GWAS*_ < 5 × 10^−8^. We detected causal effects between five vitamins and T2DM(GCST90013942). The application of the IVW method revealed that genetically predicted levels of vitamin B12 and vitamin C did not demonstrate a significant causal effect on the risk of T2DM. However, 25(OH)D (IVW *p* = 3.8 × 10^−7^, *p*_*fdr*_ = 7.6 × 10^−7^), vitamin B9 (IVW *p* = 3.9 × 10^−3^, *p*_*fdr*_ = 7.8 × 10^−3^), and vitamin B6 (IVW *p* = 7.3 × 10^−3^, *p*_*fdr*_ = 0.02) demonstrated significant causal effects on the risk of T2DM (Table 8). A 1‐SD increase in genetically predicted serum 25(OH)D levels, the odds ratio of T2DM was 0.67, 95% confidence interval:0.57–0.78 (Table 9). Subsequently, we detected causal effects between five vitamins and T2DM (GCST90029024). Our findings were consistent with previous studies. The IVW results indicated that genetically predicted levels of vitamin B12 and vitamin C did not exhibit a significant causal effect on the risk of T2DM. In contrast, significant causal effects on T2DM risk were observed for 25(OH)D (IVW *p* = 1.1 × 10^−4^, *p*_*fdr*_ = 1.1 × 10^−4^), vitamin B9 (IVW *p* = 0.02, *p*_*fdr*_ = 0.02), and vitamin B6 (IVW *p* = 0.02, *p*_*fdr*_ = 0.02) (Table 10). A 1‐SD increase in genetically predicted serum 25(OH)D levels was associated with an OR of 0.987 for T2DM with a 95% CI of 0.981–0.994 (Table 9).

**TABLE 8:** UVMR of five vitamins on T2DM (GCST90013942).

	Exposure	Method	nSNPs	*β*	Se	*p*	*p*_*fdr*_
*p*_*GWAS*_ < 5 × 10^−6^	Vitamin C	Inverse variance weighted	12	1.18	0.95	0.21	0.34
		MR Egger	12	1.32	2.13	0.55	0.69
		Weighted median	12	0.71	1.25	0.57	0.57
	Vitamin B9	Inverse variance weighted	10	6.40	2.22	3.9 × 10^−3^ *	7.8 × 10^−3^ *
		MR Egger	10	12.04	5.49	0.06	0.12
		Weighted median	10	8.99	2.66	7.3 × 10^−4^ *	1.5 × 10^−3^ *
	Vitamin B6	Inverse variance weighted	14	0.29	0.11	7.3 × 10^−3^ *	0.02*
		MR Egger	14	0.20	0.23	0.4	0.4
		Weighted median	14	0.26	0.15	0.09	0.09
*p*_*GWAS*_ < 5 × 10^−8^	Vitamin B12	Inverse variance weighted	4	< −0.01	0.04	0.93	0.93
		MR Egger	4	−0.09	0.13	0.57	0.59
		Weighted median	4	< −0.01	0.04	0.94	0.94
	25‐hydroxyvitamin D	Inverse variance weighted	141	−0.40	0.08	3.8 × 10^−7^ *	7.6 × 10^−7^ *
		MR Egger	141	−0.12	0.2	0.54	0.54
		Weighted median	141	−0.34	0.12	4.9 × 10^−3^ *	9.8 × 10^−3^ *

* *p* < 0.05, the result is statistically significant.

**TABLE 9:** UVMR of 25‐Hydroxyvitamin D on T2DM (GCST90013942 and GCST90029024).

Outcome	IVs	Method	*β*	Se	*p*	OR	95% CI	*p(fdr)*
GCST90013942	141	Inverse variance weighted	−0.40	0.08	3.8 × 10^−7^ *	0.67	0.57–0.78	7.6 × 10^−7^ *
	141	Weighted median	−0.34	0.12	4.9 × 10^−3^ *	0.71	0.56–0.90	9.7 × 10^−3^ *
	141	MR Egger	−0.12	0.19	0.54	0.89	0.61–1.30	0.54
	141	Bayesian Weighted Mendelian Randomization	−0.42	0.08	4.7 × 10^−7^ *	0.66	0.56–0.77	9.3 × 10^−7^ *
	141	Inverse variance weighted (fixed effects)	−0.40	0.08	3.8 × 10^−7^ *	0.67	0.57–0.78	7.6 × 10^−7^ *
	141	Contamination mixture method	−0.93	0.16	5.7 × 10^−7^ *	0.39	0.29–0.54	1.4 × 10^−6^ *
	141	Robust adjusted profile score (RAPS)	−0.43	0.09	5.9 × 10^−7^ *	0.65	0.55–0.77	1.2 × 10^−6^ *
	141	Debiased inverse‐variance weighted method	−0.42	0.08	4.1 × 10^−7^ *	0.66	0.56–0.77	8.3 × 10^−7^ *
	141	Constrained maximum likelihood	−0.39	0.09	3.5 × 10^−5^ *	0.67	0.56–0.81	7.0 × 10^−5^ *
GCST90029024	121	Inverse variance weighted	−0.01	< 0.01	1.1 × 10^−4^ *	0.987	0.981–0.994	1.1 × 10^−4^ *
	121	Weighted median	−0.01	< 0.01	0.07	0.998	0.982–1.001	0.07
	121	MR Egger	< −0.01	0.01	0.82	0.999	0.983–1.014	0.82
	121	Bayesian Weighted Mendelian Randomization	−0.01	< 0.01	1.5 × 10^−4^ *	0.987	0.980–0.994	1.5 × 10^−4^ *
	121	Inverse variance weighted (fixed effects)	−0.01	< 0.01	8.6 × 10^−5^ *	0.988	0.981–0.993	8.6 × 10^−5^ *
	121	Contamination mixture method	−0.04	< 0.01	1.3 × 10^−4^ *	0.964	0.959–0.969	1.3 × 10^−4^ *
	121	Robust adjusted profile score (RAPS)	−0.01	< 0.01	3.4 × 10^−4^ *	0.987	0.979–0.993	3.4 × 10^−4^ *
	121	Debiased inverse‐variance weighted method	−0.01	< 0.01	1.0 × 10^−4^ *	0.987	0.981–0.994	1.0 × 10^−4^ *
	121	Constrained maximum likelihood	−0.01	< 0.01	3.2 × 10^−4^ *	0.987	0.980–0.995	3.2 × 10^−4^ *

* *p* < 0.05, the result is statistically significant.

**TABLE 10:** UVMR of five vitamins on T2DM (GCST90029024).

	Exposure	Method	nSNPs	*β*	Se	*p*	*p*_*fdr*_
*p*_*GWAS*_ < 5 × 10^−6^	Vitamin C	Inverse variance weighted	17	1.18	0.95	0.34	0.34
		MR Egger	17	1.32	2.13	0.69	0.69
		Weighted median	17	0.71	1.25	0.36	0.57
	Vitamin B9	Inverse variance weighted	11	0.2	0.08	0.02*	0.02*
		MR Egger	11	0.31	0.21	0.18	0.18
		Weighted median	11	0.14	0.11	0.19	0.19
	Vitamin B6	Inverse variance weighted	16	0.29	0.11	0.02*	0.02*
		MR Egger	16	0.20	0.23	0.32	0.39
		Weighted median	16	0.26	0.15	0.05*	0.09
*p*_*GWAS*_ < 5 × 10^−8^	Vitamin B12	Inverse variance weighted	5	< 0.01	< 0.01	0.18	0.35
		MR Egger	5	< −0.01	0.01	0.59	0.59
		Weighted median	5	< 0.01	< 0.01	0.08	0.17
	25‐Hydroxyvitamin D	Inverse variance weighted	121	−0.01	< 0.01	1.1 × 10^−4^ *	1.1 × 10^−4^ *
		MR Egger	121	< −0.01	0.01	0.82	0.82
		Weighted median	121	−0.01	0.01	0.07	0.07

* *p* < 0.05, the result is statistically significant.

Genetically predicted serum 25(OH)D concentrations exhibit a significant association with T2DM in both MVMR and UVMR analyses. Furthermore, we employed a variety of alternative methods for MR analysis. Specifically, the CML‐MA (Constrained Maximum Likelihood) method was applied to mitigate bias arising from both related and unrelated pleiotropy [47]. The ConMix (Containment Mixture) method was also utilised, which aids in identifying groups of genetic variants with similar causal estimates [48]. These groups may signify distinct mechanisms by which risk factors influence the outcomes. The ConMix method demonstrates robust and effective performance in MR even when confronted with invalid IVs. When compared to other robust methods, it exhibits the lowest mean square error across a range of real‐world scenarios. The Robust Adjusted Profile Score (MR‐RAPS) method facilitates the inclusion of some weak IVs, thereby enabling robust statistical estimation in MR [49]. The Debased inverse variance weighted (dIVW) method is a modified IVW method that mitigates the weak instrument bias inherent in the traditional IVW method, offering enhanced robustness in the presence of numerous weak instruments [50]. Bayesian weighted Mendelian randomization (BWMR) method serves as a valuable method for elucidating the causal relationships between risk factors (exposures) and complex traits or diseases (outcomes) [51]. Notably, SNPs associated with serum 25(OH)D concentrations have demonstrated a negative correlation with the risk of T2DM (GCST90013942), including a specific association with BWMR (*p* = 4.7 × 10^−7^, *p*_*fdr*_ = 9.3 × 10^−7^), Commix (*p* = 5.7 × 10^−7^, *p*_*fdr*_ = 1.4 × 10^−6^), MR‐RAPS (*p* = 5.9 × 10^−7^, *p*_*fdr*_ = 1.2 × 10^−6^), IVW fixed effects method (*p* = 3.8 × 10^−7^, *p*_*fdr*_ = 7.6 × 10^−7^), dIVW (*p* = 4.1 × 10^−7^, *p*_*fdr*_ = 8.3 × 10^−7^), CML‐MA (*p* = 3.5 × 10^−5^, *p*_*fdr*_ = 7.0 × 10^−5^) (Table 9). Furthermore, the negative correlation of serum 25(OH)D levels with T2DM risk was corroborated by the findings of GCST90029024. Significant correlations were found between BWMR (*p* = 1.5 × 10^−4^, *p*_*fdr*_ = 1.5 × 10^−4^), Conmix (*p* = 1.3 × 10^−4^, *p*_*fdr*_ = 1.3 × 10^−4^), MR‐RAPS (*p* = 3.4 × 10^−4^, *p*_*fdr*_ = 3.4 × 10^−4^), dIVW (*p* = 1.0 × 10^−4^, *p*_*fdr*_ = 1.0 × 10^−4^), IVW (fixed effects) (*p* = 8.6 × 10^−5^, *p*_*fdr*_ = 8.6 × 10^−5^), CML‐MA (*p* = 3.2 × 10^−4^, *p*_*fdr*_ = 3.2 × 10^−4^) (Table 9). Given the presence of two T2DM outcomes, we applied the False Discovery Rate (FDR) correction to adjust our results once more. We observed that the *P*‐values of the MR‐Egger method for analysing serum 25(OH)D concentrations in relation to two distinct outcomes of T2DM were not statistically significant. These lacks of significance may be attributed to insufficient statistical power. The MR‐Egger method is particularly sensitive to outlier SNPs and exhibits a greater standard deviation compared to alternative methods. Furthermore, there may be a lack of detectable pleiotropy.

**FIGURE 3:**
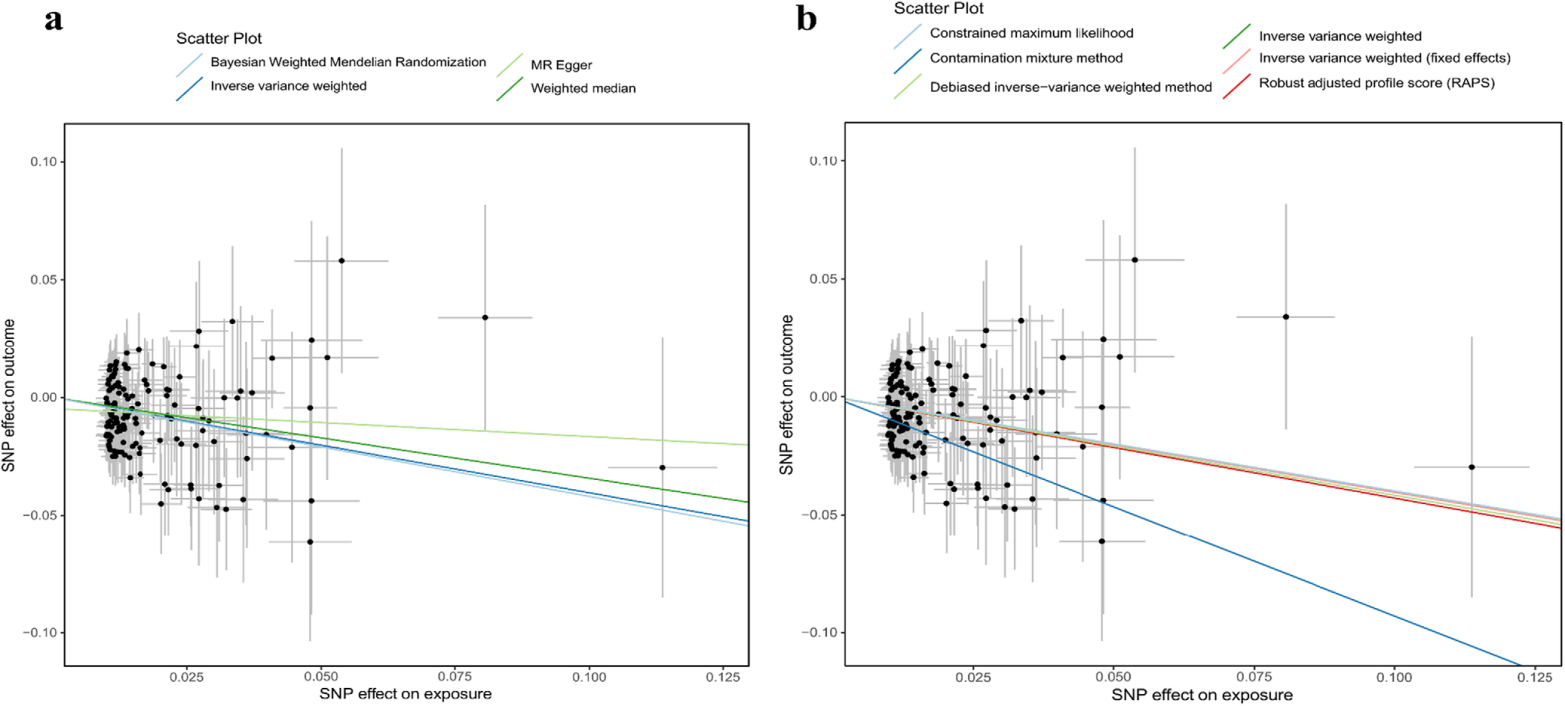
Scatter plot of 25‐hydroxyvitamin D and T2DM (GCST90013942). (a) present scatter plots for 4 MR methods, including Inverse Variance Weighted, Weighted Median, MR Egger, and Bayesian Weighted Mendelian Randomization. (b) present scatter plots for 5 MR methods, including Inverse Variance Weighted (Fixed Effects), Contamination Mixture Method, Robust Adjusted Profile Score (RAPS), Debiased Inverse‐Variance Weighted Method, and Constrained Maximum Likelihood.

**FIGURE 4:**
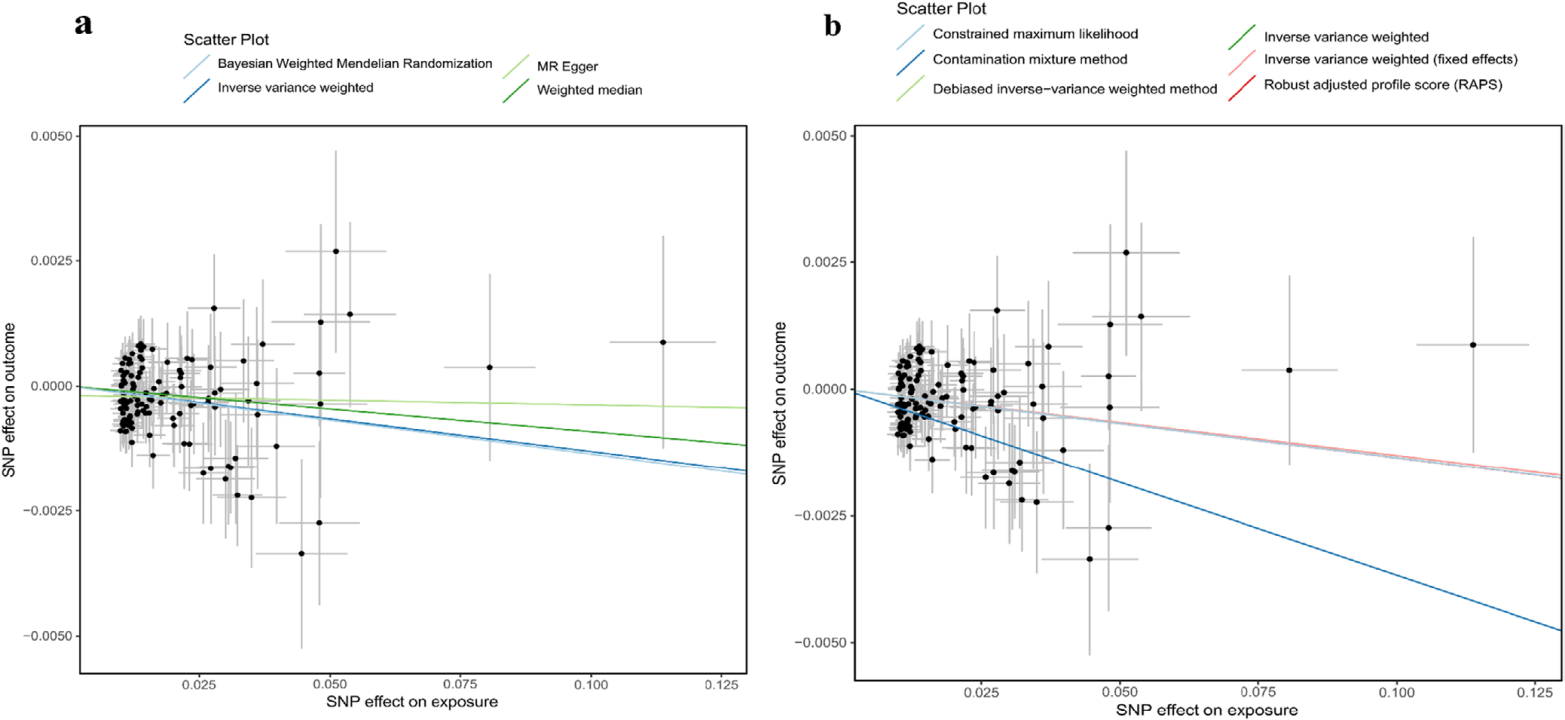
Scatter plot of 25‐hydroxyvitamin D and T2DM (GCST90029024). (a) present scatter plots for 4 MR methods, including Inverse Variance Weighted, Weighted Median, MR Egger, and Bayesian Weighted Mendelian Randomization. (b) present scatter plots for 5 MR methods, including Inverse Variance Weighted (Fixed Effects), Contamination Mixture Method, Robust Adjusted Profile Score (RAPS), Debiased Inverse‐Variance Weighted Method, and Constrained Maximum Likelihood.

We conducted analyses to assess heterogeneity, pleiotropy, and sensitivity. After excluding the outliers identified through the MR‐PRESSO analysis, we derived estimates that aligned with our initial findings, thereby confirming the robustness of our results following adjustments for pleiotropic effects. Moreover, our examination utilising steiger test indicated the absence of a reverse causal relationship among the identified SNPs, and the *F*‐statistic reinforced the reliability of the inferred causal direction. We evaluated the potential influence of pleiotropy through the MR‐Egger intercept and the MR‐PRESSO Global test (Tables 3 and 5). The heterogeneity test (Cochran's *Q* test) is shown in Tables 2 and 4. The leave‐one‐out analysis conducted by sequentially excluding individual SNPs demonstrated no substantial alteration in effect estimation (Figures S1 and S2). Figure 3 presents the scatter plot of 25(OH) D and T2DM (GCST90013942), while Figure 4 illustrates the scatter plot of 25(OH)Dand T2DM (GCST90029024). The scatter plot primarily assesses the validity of the IVs. The negative slopes of several linear regression lines suggested a negative correlation between serum 25(OH)D levels and T2DM (Figures 3 and 4).

## 4 Discussion

Using a comprehensive analytical method, we investigated serum 25(OH)D levels in relation to T2DM. The findings from our UVMR and MVMR analyses suggest that higher vitamin D status [serum 25(OH)D] reduces the risk of the development of T2DM.

Vitamin D has been suggested as a protective compound for T2DM. Several mechanisms linking vitamin D to the regulation of the immune response support its role for vitamin D in the pathogenesis of T2DM. Firstly, the active form of vitamin D, 1,25‐dihydroxyvitamin D, is pivotal in the regulation of insulin synthesis and secretion. It binds to the vitamin D receptor (VCR), which subsequently induces the expression of a series of genes associated with glucose transport and insulin secretion [52, 53]. Secondly, vitamin D indirectly modulates insulin secretion by influencing intracellular calcium concentrations. The elevation in intracellular calcium concentration enhances insulin secretion. Furthermore, vitamin D regulates intracellular calcium concentration, encompassing the activation of protein kinase A (PKA) and the enhancement of L‐type voltage‐dependent Ca^2+^ channels to promote insulin secretion [54]. Moreover, vitamin D modulates the expression of voltage‐gated calcium channels through the activation of the vitamin D receptor (VDR), thereby enhancing intracellular calcium concentration and promoting insulin secretion [54]. Considering the substantial body of existing evidence supporting the essential roles of higher vitamin D status [serum 25(OH)D levels] in reducing insulin resistance and maintaining adequate insulin secretory responses, and causing prospective reductions in T2DM risk with higher baseline vitamin D levels. It is noteworthy to mention the findings from the D2d (vitamin D and T2DM) trial, which domenstrated a significant reduction in the risk of T2DM through supplementation that achieves serum 25(OH)D values of 100 nmol/L or higher in individuals with pre‐diabetes [55].

Serum 25(OH)D levels have been inversely correlated with the risk of T2DM. Over a 29‐year follow‐up period, a cohort study of 9841 people showed that low serum 25(OH)D levels were related to an increased risk of T2DM, regardless of sex age, body mass index (BMI), and other health‐related factors [56]. 1572 incident cases of T2DM have been identified in an observational study involving 53,088 participants over a mean follow‐up period of 6.6 years. The study examined the relationship between serum 25(OH)D levels and incident cases of T2DM [57]. The results of another observational study examining vitamin D levels and diabetic foot complications found that higher vitamin D levels were associated with a reduced risk of diabetic foot complications [58]. A Norwegian study showed that serum 25(OH)D < 50 nmol/L was associated with an increased risk of T2DM in Norwegian adults [59]. A comprehensive meta‐analysis was conducted, incorporating data from 21 prospective studies with a cumulative sample size of 76,220 participants and 4996 incident cases of T2DM. The impact of vitamin D supplementation on glycemic control in patients with T2DM was examined in a systematic review and meta‐analysis. The findings indicated that oral vitamin D supplementation significantly improved serum 25(OH)D status and reduced insulin resistance compared to placebo treatments in this patient population [60]. A bidirectional MR study indicates that genetically instrumented risk for T2DM may not lead to alterations in serum 25(OH)D levels. However, a deficiency in genetically regulated 25(OH)D, attributable to the vitamin D synthesis gene DHCR7, may affect the risk of developing T2DM [61]. A nonlinear MR study investigated the association between vitamin D deficiency and increased mortality risk within the UK Biobank. This research established causality for various health outcomes, including cardiovascular disease, dementia, T2DM, and all‐cause mortality rates [62].

## 5 Strengths and Limitations

Our study possesses several strengths and limitations. Based on data from a large‐scale GWAS, it is the first study to investigate the causal relationship between five specific vitamins and T2DM using the MR method. The MR method offers a notable advantage through its implementation. Thus, causal inferences are strengthened by reducing residual confounding and other biases. The UVMR and MVMR employed in this study surpass prior observational research due to the utilisation of summary data from GWAS, characterised by an extensive sample size and a substantial number of SNPs. Furthermore, our findings demonstrate robustness and reliability, exhibiting no evidence of heterogeneity or pleiotropic effects. Nonetheless, our study has certain inherent limitations. Primarily, the genetic variation data mainly come from GWAS data involving individuals of European descent, potentially limiting the generalisability of our results to more diverse populations. However, limiting the lineage of participants serves to reduce potential confounding effects associated with population admixture. Moreover, we are unable to identify the mechanisms behind the observed correlations since we do not have the ability to assess nonlinear relationships. Additionally, we cannot detect thresholds or saturations because we rely on summary level data.

## 6 Conclusion

Our MR study indicates that higher vitamin D status [serum 25(OH)D levels] is associated with a reduced risk of developing T2DM. These findings carry significant clinical implications, suggesting that enhancing vitamin D levels could contribute to the prevention of T2DM in European populations.

## Author Contributions

Jin Yang participated in investigations, data search, analysis methods, data analysis, chart drawing, writing, editing, reviewing and correspondence.

## Conflicts of Interest

The author declares no conflicts of interest.

## Supporting information


Figure S1.

Table S1.

Table S2.

Table S3.

Table S4.

Table S5.

Table S6.

## Data Availability

The data that support the findings of this study are available on request from the corresponding author. The data are not publicly available due to privacy or ethical restrictions.
