# Supplementary material for: Genetically Predicted Serum 25‐Hydroxyvitamin D Concentrations in Related to Type 2 Diabetes Mellitus: A Mendelian Randomization Study
Source: Endocrinol Diabetes Metab. 2025 Jul 9;8(4):e70050. doi: 10.1002/edm2.70050 (PMC12240638; doi:10.1002/edm2.70050)
Supplement: Supplementary file 1 — Figure S1. [file EDM2-8-e70050-s006.docx]

**Supplementary Figure 1**

**
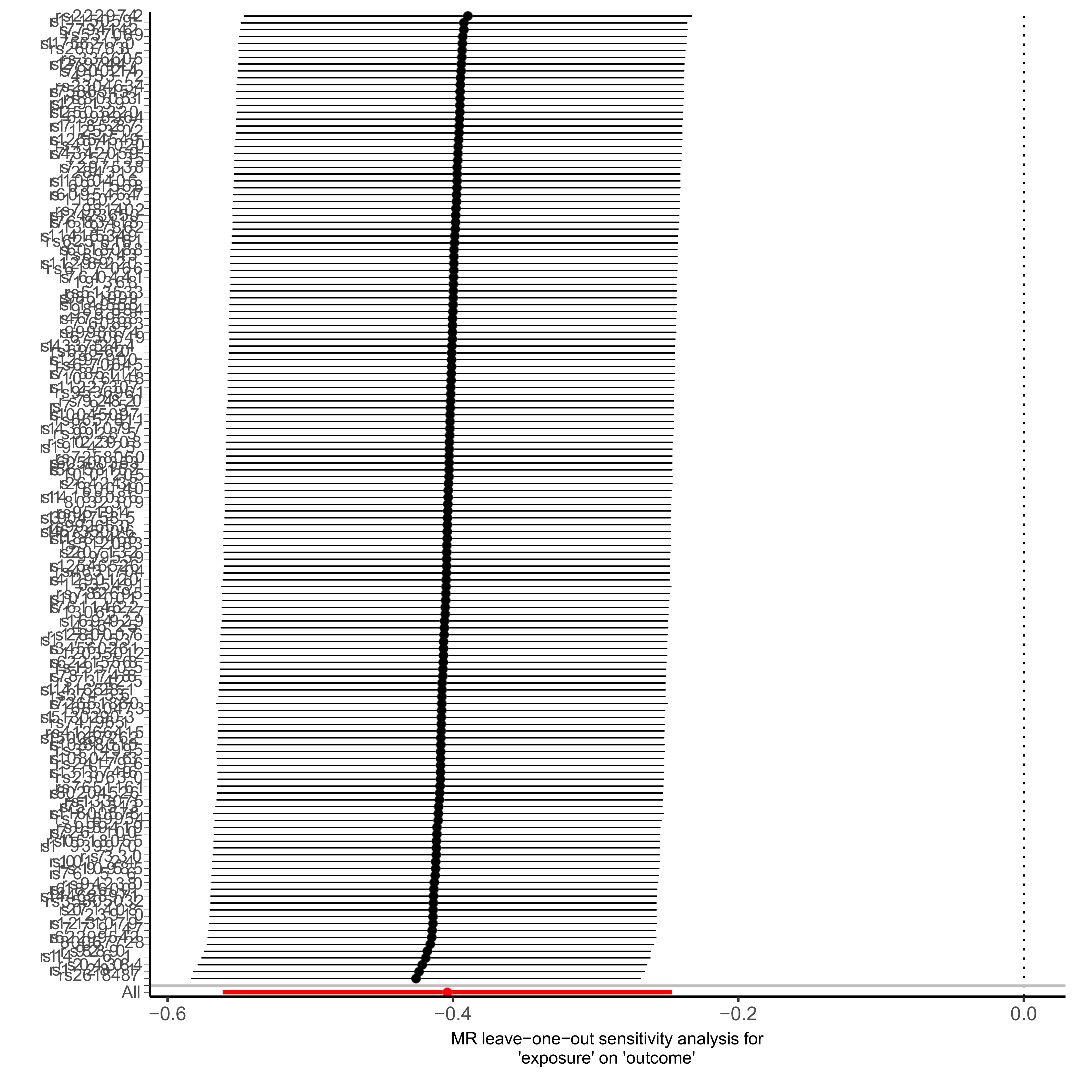
**

**Supplementary Figure 1 Leave-one-out plot of 25-hydroxyvitamin D and T2DM (GCST90013942)**

**Supplementary Figure 2**

**
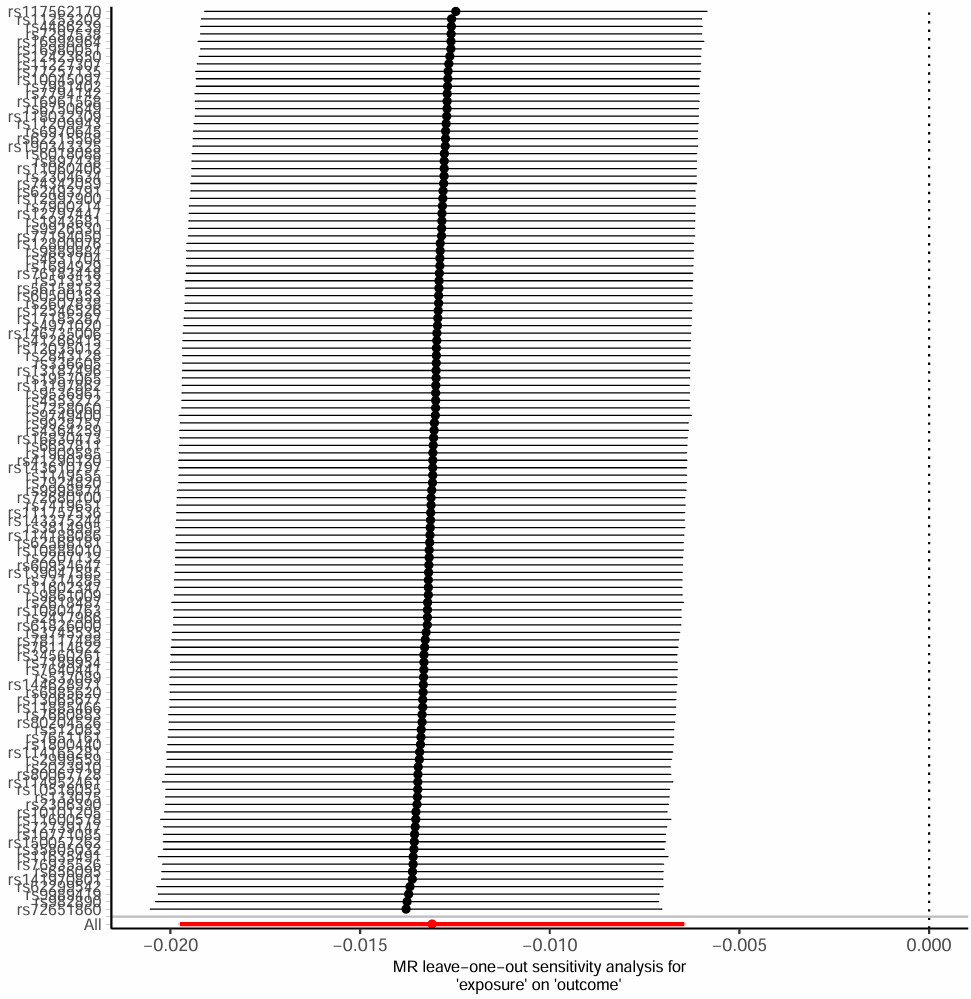
**

**Supplementary Figure 2. Leave-one-out plot of 25-hydroxyvitamin D and T2DM (GCST90029024)**
